# Multi-Domain Screening: Identification of Patient’s Risk Profile Prior to Head-and-Neck Cancer Treatment

**DOI:** 10.3390/cancers15215254

**Published:** 2023-11-01

**Authors:** Monse W. M. Wieland, Walmari Pilz, Bjorn Winkens, Ann Hoeben, Anna C. H. Willemsen, Bernd Kremer, Laura W. J. Baijens

**Affiliations:** 1 Department of Otorhinolaryngology, Head and Neck Surgery, Maastricht University Medical Center, 6202 AZ Maastricht, The Netherlands; 2 GROW-School for Oncology and Reproduction, Maastricht University, 6229 ER Maastricht, The Netherlands; 3 Department of Methodology and Statistics, Maastricht University, 6200 MD Maastricht, The Netherlands; 4 Care and Public Health Research Institute—CAPHRI, Maastricht University, 6200 MD Maastricht, The Netherlands; 5 Division of Medical Oncology, Department of Internal Medicine, Maastricht University Medical Center, 6202 AZ, The Netherlands; 6 Department of Internal Medicine, Diakonessenhuis, 3508 TG Utrecht, The Netherlands; 7 Department of Otorhinolaryngology and Head and Neck Surgery, Erasmus MC Cancer Institute, University Medical Center Rotterdam, 3000 CA Rotterdam, The Netherlands

**Keywords:** head and neck cancer, screening, oropharyngeal dysphagia, malnutrition, sarcopenia, frailty

## Abstract

**Simple Summary:**

Oropharyngeal dysphagia (OD), malnutrition, sarcopenia, and frailty often co-occur with head-and-neck cancer (HNC) and may affect treatment outcomes, but the presence, severity, and consequences of these phenomena vary from patient to patient. It is a challenge to predict which patients have a higher risk of these phenomena, and early identification using a ‘quick and easy’ multi-domain screening may allow us to obtain a more holistic view of the patient’s risk profile, enabling the prevention of complications and prehabilitation before the start of cancer treatment. The aim of our study was to identify the prevalence of the risk of OD, malnutrition, sarcopenia, and frailty and their co-occurrence in all newly diagnosed HNC patients. More than three quarters of the 128 patients were at risk for OD, malnutrition, sarcopenia, and/or frailty. The advanced cancer stage was related to an increased risk of OD and higher levels of distress.

**Abstract:**

Background: Head-and-neck cancer (HNC) can give rise to oropharyngeal dysphagia (OD), malnutrition, sarcopenia, and frailty. Early identification of these phenomena in newly diagnosed HNC patients is important to reduce the risk of complications and to improve treatment outcomes. The aim of this study was (1) to determine the prevalence of the risk of OD, malnutrition, sarcopenia, and frailty; and (2) to investigate the relation between these phenomena and patients’ age, performance status, and cancer group staging. Methods: Patients (N = 128) underwent multi-domain screening consisting of the Eating Assessment Tool-10 for OD, Short Nutritional Assessment Questionnaire and BMI for malnutrition, Short Physical Performance Battery and Hand Grip Strength for sarcopenia, and Distress Thermometer and Maastricht Frailty Screening Tool for frailty. Results: 26.2%, 31.0%, 73.0%, and 46.4% of the patients were at risk for OD, malnutrition, sarcopenia, or frailty, respectively. Patients with an advanced cancer stage had a significantly higher risk of OD and high levels of distress prior to cancer treatment. Conclusions: This study identified the risk profile of newly diagnosed HNC patients using a standardized ‘quick and easy’ multi-domain screening prior to cancer treatment.

## 1. Introduction

Head and neck cancer (HNC) is a collective term for a group of malignant tumors that arise in the oral cavity, nasal cavity, paranasal sinuses, pharynx, larynx, or salivary glands [1]. In 2019, approximately 3000 new HNC cases were registered in the Netherlands [2], and in 2022, 625 new cases were registered in the Comprehensive Cancer Center of Maastricht University Medical Center+ (MUMC+). 

Oropharyngeal dysphagia (OD), malnutrition, sarcopenia, and frailty often co-occur with HNC and may influence the choices and outcomes of cancer treatment [3,4,5,6,7]. The relationship between HNC and these phenomena is not always predictable or straightforward, as their presence, severity, and consequences may vary. For instance, in some cases, swallowing function is affected by primary tumor-related obstruction of the upper digestive tract [8]. However, small primary tumors with extensive lymph node metastases that invade important swallowing structures such as cranial nerves and muscles can also lead to OD [9,10]. In turn, OD can affect oral intake, which, if not diagnosed early, can lead to malnutrition and loss of skeletal muscle mass, further promoting OD [10]. This is an example of how these phenomena interrelate. Pre-existing malnutrition is common in HNC patients, usually due to an unhealthy lifestyle with excessive tobacco and alcohol consumption and a diet lacking various essential nutrients like vitamins, minerals, proteins, fibers, etc. [11,12]. Moreover, the nutritional status of HNC patients can also be affected by the cancer metabolism itself [13]. Inadequate nutritional intake leading to poor nutritional status is one of the mechanisms involved in the onset of sarcopenia, a syndrome characterized by ongoing loss of skeletal muscle mass and strength resulting in physical disability [14,15]. Sarcopenia is highly prevalent in newly diagnosed HNC patients, ranging from 6.6 to 64.6% [16]. Besides the increased risk of OD, malnutrition, and sarcopenia, HNC patients have an increased risk of being ‘frail’ [17]. Frailty is defined as a patient’s increased vulnerability to stressors due to the lack of physiological reserves [18]. Age is an important aspect to consider when investigating frailty in HNC. The prevalence of frailty is known to increase with age [18]; the HNC population is mainly made up of patients over 60 years of age, and approximately one-fourth of patients are 75 years or older [19,20]. Besides age, comorbidities such as congestive heart failure, diabetes, hypertension, and chronic obstructive pulmonary disease are highly related to frailty and to an unhealthy lifestyle [18,21,22]. 

It is a challenge for the interdisciplinary team to distinguish between patients with HNC who can or cannot sustain adequate cancer treatment in terms of comorbidity and frailty. Early identification and treatment of OD, malnutrition, sarcopenia, and frailty in newly diagnosed HNC patients is crucial to prepare patients for cancer treatment, lower the risk of complications and toxicity, and improve oncological outcomes such as survival and health-related quality of life [17,23,24]. The purpose of screening is to identify HNC patients who are at risk for a particular condition and may need additional diagnostics and treatment [25]. 

Currently, studies reporting on multiple comorbid phenomena in newly diagnosed HNC patients are usually based on multi-domain diagnostic assessment methods, such as the comprehensive geriatric assessment (CGA) being considered the gold standard to identify medical, psychosocial, and functional limitations of a frail elderly person [26,27,28]. However, there is no consensus on which tools to include in a CGA [29]. Furthermore, a CGA is usually carried out by a multi or interdisciplinary team. It is time consuming—between 80 and 120 min—for the patient and the health professionals, and this limits its use in everyday practice [29,30]. The time required for multi-domain screening is likely to be shorter.

Previous studies have already reported on single-domain screening or the diagnostic assessment of either OD, malnutrition, sarcopenia, or frailty prior to HNC treatment [7,31,32,33,34,35,36,37]. However, to our knowledge, no studies have been published on multi-domain screening for OD, malnutrition, sarcopenia, and frailty at the same time in newly diagnosed HNC patients. Therefore, the prevalence of the risk of OD, malnutrition, sarcopenia, and frailty and their co-occurrence remains unknown. Multi-domain screening allows us to obtain a more holistic view of the patient’s risk profile, which can benefit a patient-centered approach to cancer treatment, prehabilitation, and rehabilitation.

Age, performance status (PS), and cancer stage grouping are major criteria used in cancer treatment decision-making as they are important predictors of tolerance to treatment and survival [38,39,40]. To optimize the patient-centered approach to cancer treatment in HNC patients, it is interesting to understand the relationship between patients’ age, PS, and cancer stage grouping versus the risk of OD, malnutrition, sarcopenia, and frailty. As stated above, the presence, severity, and consequences of OD, malnutrition, and sarcopenia may vary in HNC patients, and these phenomena are likely to interrelate and affect cancer treatment outcomes. The relationship between age and PS in HNC has previously been described [41]. Frailty is associated with higher age, and one of the dominant clinical presentations of frailty is fluctuation in functional ability [18]. However, no previous studies have examined the association between age, PS, and cancer stage grouping versus the risk of OD, malnutrition, sarcopenia, and frailty by screening all newly diagnosed HNC patients. The understanding of this relationship can guide us to more effective screening and management of a patient’s risk profile prior to cancer treatment. 

It is hypothesized that newly diagnosed HNC patients are at risk of OD, malnutrition, sarcopenia, and frailty and that a higher risk in these four domains may be associated with higher age, poor PS, and advanced cancer stage grouping. Therefore, the aim of this study is two-fold: (1) to determine the prevalence of the risk of OD, malnutrition, sarcopenia, and frailty at the same time in newly diagnosed HNC patients, and (2) to investigate the relationship between patients’ age, PS, and cancer stage grouping on one hand versus the risk of OD, malnutrition, sarcopenia, and frailty on the other hand. 

## 2. Materials and Methods

### 2.1. Study Design and Study Population

The baseline data of a prospective cohort study were analyzed to address the research questions. The study took place in the Comprehensive Cancer Center of Maastricht University Medical Center+ (MUMC+) in the Netherlands and was approved by the medical ethics committee (METC 2022-3133) according to the non-WMO (Wet Medisch-Wetenschappelijk Onderzoek) obligatory Medical Research Involving Human Subjects Act [42]. Patients gave their informed consent upon participation. The study was conducted in accordance with the Declaration of Helsinki.

Patients were enrolled in the study if they were newly diagnosed with HNC between December 2021 and July 2022. Exclusion criteria were those younger than 18 years, not being able to give informed consent; illiteracy or blindness; not having undergone the multi-domain screening; having a diagnosis of incurable HNC; or a diagnosis other than HNC such as sarcoma, carcinoma of the thyroid gland, skin cancer, and hematological malignancies. 

### 2.2. Demographic and Oncological Data Collection

In 2014, the Dutch Head and Neck Society (DHNS) started a prospective audit on a national level, the Dutch Head and Neck Audit (DHNA), to monitor and measure the quality of HNC care [43]. The DHNA is a mandatory quality health care registration system that contributes to standardized and strictly protocolled data registration [44]. All outcome indicators used in this system are evidence-based. The demographic and oncological data of the current study was extracted from the DHNA records by the first author in a standardized way. The data was randomly checked by the second author for accuracy. Data on patient demographics (age; sex; tobacco and alcohol consumption; marital status; and occupation), tumor characteristics (primary tumor site; tumor, nodes, and metastasis (TNM) classification; cancer stage grouping; and histopathological diagnosis), comorbidity, and PS were collected from the DHNA records. In the final DHNA, TNM classification and cancer stage grouping are carried out during the interdisciplinary tumor board meetings according to the TNM classification 8th edition [45]. Comorbidity was scored using the Charlson Comorbidity Index (CCI) grade [46]. This validated tool uses International Classification of Diseases (ICD-10) data to score comorbidity, and CCI grades range from 0 to 3, where “0” represents no comorbid and “3” represents a severe comorbid condition. The patient’s ability to independently perform activities of daily living (ADL) was assessed using the PS according to the World Health Organization (WHO) scale [47]. This ordinal scale ranges from 0 to 4, where “0” represents a fully active condition and “4” represents completely disabled. 

### 2.3. Multi-Domain Screening

Consecutive newly diagnosed HNC patients underwent a standardized multi-domain screening protocol within 10 days after cancer diagnosis. The protocol consists of screening instruments for the risk of OD, malnutrition, sarcopenia, and frailty and is described below. 

The multi-domain screening was performed by three oncology nurses with more than 10 years of clinical experience in HNC. Prior to the introduction of the screening protocol in everyday practice, the nurses followed intensive multiple-session training on the purpose of the screening and how to carry out the measurements in a standardized way. The nurses were randomly supervised during screening by the first author to guarantee continuous standardized data collection.

The Eating Assessment Tool (EAT-10) is a patient-reported dysphagia-specific symptom questionnaire consisting of 10 items, and the answers are based on a 5-point Likert scale ranging from “0” representing no swallowing problem to “4” representing severe swallowing problems [48,49]. The maximum total score is 40 points, and a score of ≥3 indicates a high risk of self-perceived OD. 

The Short Nutritional Assessment Questionnaire (SNAQ) and the Body Mass Index (BMI) were used to identify the risk of malnutrition according to the recommendation of the Dutch clinical practice guideline of malnutrition [50,51]. The validated Dutch version of the SNAQ is a patient-reported questionnaire and consists of four items covering unintentional weight loss, reduced appetite, and the use of supplemental drinks or tube feeding [50]. The maximum total score is 7 points, and a score of ≥2 indicates a high risk of malnutrition. BMI was calculated based on weight and height. Patients with a BMI < 20 kg/m^2^ and <70 years of age, or <22 kg/m^2^ and ≥70 years of age, were identified as being at risk of malnutrition, even if the SNAQ score was <2 [15,51]. 

The risk of sarcopenia was measured using the Short Physical Performance Battery (SPPB) and Hand Grip Strength (HGS) [15]. According to the European Working Group on Sarcopenia in Older People (EWGSOP2), sarcopenia is defined as a progressive loss of skeletal muscle function that is diagnosed by detecting low muscle quantity, quality, and strength, and the severity of sarcopenia correlates with increased physical disability [15]. Patients’ lower extremity function and strength were assessed using the SPPB [52,53,54]. This is a performance test to determine the risk of physical disability. Measurements were performed according to the standard operating procedure provided by the American National Institute on Aging [55]. The SPPB consists of three subtests: a balance test, a 4 m walking test, and a chair stand test [52,53]. The maximum total score is 12 points. A score between 4 and 9 points indicates a high risk of physical disability, and a score <4 indicates a severe risk of physical disability. For HGS, isometric muscle contraction of the hand was measured, and this is an indicator of overall muscle strength [56]. The JAMAR^®^ Hydraulic Hand Dynamometer was used in this cohort (Performance Health Supply Inc., Cedarburg, WI, USA). The HGS measurements were performed three times on each side (left and right hand) according to the standard operating procedure of the Dutch Nutritional Assessment Platform [57]. The highest value of the three measurements of the dominant hand was used for data analysis. Values were compared with sex-, age-, and height-appropriate references for healthy persons [58]. An HGS lower than the 10th percentile (<10th percentile) indicates a high risk of low muscle strength [58]. 

The distress thermometer (DT) and the Maastricht Frailty Screening Tool for hospitalized patients (MFST-HP) were used to identify the risk of frailty [59,60,61]. The validated DT is a patient-reported questionnaire and consists of a visual analog scale and a 46-item problem list about sources of distress that cancer patients may experience in the week prior to screening [61]. The DT visual analog scale ranges from “0” representing no distress to “10” representing extreme distress. The 46-item problem list covers practical, family/social, emotional, religious/spiritual, and/or physical problems. Patients who scored ≥5 points on the visual analog scale were identified as being at risk of presenting high levels of distress. The validated MFST-HP is a clinician-reported questionnaire and consists of 15 items covering three domains: physical, psychological, and social. The MFST-HP is validated for patients older than 70 years. Patients younger than 70 with a high-comorbid condition underwent an MFST-HP as well. Each item of the MFST-HP scores one point. A total score ≥ 6 points indicates a high risk of frailty. 

### 2.4. Statistical Analysis 

Numerical variables were reported as means with standard deviations (SDs) and medians with interquartile ranges (IQR, 25th to 75th percentile) for normally and not normally distributed characteristics, respectively, where normality was checked using histograms and Q-Q plots. Categorical variables were reported in terms of numbers and percentages. Age was initially reported as a numerical variable. Scores of the PS and cancer stage grouping were initially descriptively reported using their full range of categories: “0”, “1”, “2”, “3”, and “4” for PS, and “0–1”, “2”, “3”, and “4” for cancer stage grouping. To increase the number of patients per category, age, and PS scores were subsequently pooled into two categories, age 70 years or younger versus age above 70, and category 0–1 representing independent to perform ADL versus category 2–3–4 representing dependent to perform ADL, respectively. A PS cut-off value ≥ 2 was used as this value is associated with worse cancer treatment outcomes compared to PS 0–1 [62,63]. Also, cancer stage grouping scores were dichotomized into two categories, with categories 0–2 representing tumors localized to the organ of origin (early-stage disease) versus categories 3–4 representing local to distant widespread tumors (advanced-stage disease). Group comparisons between the dichotomized age, PS, and cancer stage grouping were performed using chi-square, Fisher’s exact test, independent samples *t*-test, and the Mann–Whitney U-test, where appropriate. After that, multivariable logistic regression analysis was performed whereby multiple models were created in which the association between age and cancer stage grouping versus increased risk of OD, malnutrition, sarcopenia, and frailty was analyzed for each outcome separately. In these models, additional correction was carried out for tobacco and alcohol consumption. For multivariable logistic regression analysis, the variables on tobacco and alcohol consumption were dichotomized into two categories: “current smoker and/or drinker” versus “no or former smoker and/or drinker”. Also, the scores of all multi-domain screening variables were dichotomized based on their validated cut-off values. Dichotomizing variables can lead to overly optimistic research results, loss of information, lower measurement accuracy, and loss of power in the analyses. Therefore, these outcome variables were also analyzed using the full range of their scores in a sensitivity analysis (linear regression analysis) to see if the dichotomization resulted in a loss of information. Multicollinearity was defined as variance inflation factor (VIF) ≥ 10, while a Cook’s distance ≥1 indicated an influential outlier. Because of the low subgroup size of the dichotomized PS category 2–3–4 (N = 14), PS was not included in the multivariable logistic regression analysis. For the same reason, HGS and MFST-HP were not included in the multivariable logistic regression analysis (HGS < 10th percentile N = 9 and MFST-HP ≥ 6 N = 4, respectively). Statistical significance was defined as two-sided *p* ≤ 0.05. Statistical analyses were performed using IBM SPSS statistics v.28 (IBM SPSS Statistics, IBM Corporation, Armonk, NY, USA). 

## 3. Results

### 3.1. Screening Participation Rates

**Figure 1:**
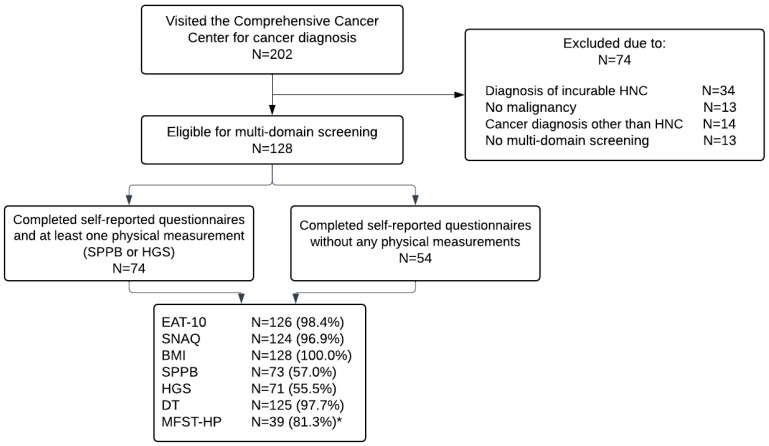
Flowchart of patients’ enrollment. Abbreviations: N—Number of patients; SPPB—Short Physical Performance Battery; HGS—Hand Grip Strength; EAT-10—Eating Assessment Tool; SNAQ—Short Nutritional Assessment Questionnaire; BMI—Body Mass Index; DT—Distress thermometer; MFST-HP—Maastricht Frailty Screening Tool. * 39 patients out of N = 48 (only patients aged above 70 years and patients younger than 70 with a severe comorbid condition).

Of the 202 patients who visited the Comprehensive Cancer Center between December 2021 and July 2022, 128 (63.4%) met the inclusion criteria. Of the included patients, 74 (57.8%) patients completed the self-reported questionnaires and at least one physical measurement (SPPB or HGS), and 54 (42.2%) patients completed the self-reported questionnaires without any physical measurement. The reasons for missing data on physical measurements were multiple, including patients’ fear of contracting COVID-19, fatigue, lack of time for oncology nurses to perform the screening, schedule conflicts with other cancer diagnostic procedures, and patients’ refusal without explanation. A flowchart of the patient enrollment is shown in Figure 1. 

### 3.2. Patient Demographic Data

The mean age of the included patients was 67 (SD 9.8), and 84 (65.6%) patients were male. The oral cavity was the most common primary tumor site (N = 46; 35.9%). In 115 (89.8%) patients, the histopathological diagnosis was squamous cell carcinoma. Seventy-six (59.4%) patients had a CCI grade of ≥1, and thirty-eight (29.7%) patients had a PS score ≥ 1, indicating that comorbid conditions were quite prevalent. An overview of patient demographic data, tumor characteristics, CCI grades, and PS scores are presented in Table 1.

### 3.3. Prevalence of the Risk of OD, Malnutrition, Sarcopenia, and Frailty 

Of the 128 patients, 94 (73.4%) were at risk of at least one of the four screening domains. A total of 33 out of 126 (26.2%) patients were at risk of OD (EAT-10 ≥ 3), and 39 out of 126 (31.0%) patients were at risk of malnutrition (SNAQ ≥ 2 or BMI < 20 kg/m^2^ if age < 70 y and <22 kg/m^2^ if age ≥ 70 y). The risk of sarcopenia (SPPB ≤ 9 or HGS < 10th percentile) was identified in 54 out of 74 (73.0%) patients. A total of 58 out of 125 (46.4%) patients presented high levels of distress (DT ≥ 5), and 4 out of 39 (10.3%) patients presented a high risk of frailty (MFST-HP ≥ 6). 

The frequency distribution, measures of central tendency, and dispersion of the multi-domain screening are given in Table 2.

### 3.4. Relationship between Age, PS, and Cancer Stage Grouping Versus Risk of OD, Malnutrition, Sarcopenia, and Frailty

Univariable linear regression analysis did not show a significant association between age and the dichotomized screening outcomes of risk of OD, malnutrition, sarcopenia, and risk of frailty. However, median SNAQ and SPPB scores significantly differed between age group ≤ 70 versus >70 (Table 3). 

No significant associations were found between PS (2–3 versus 0–1) versus risk of OD, malnutrition, and high levels of distress. On the other hand, HNC patients with a poor PS (2–3) had a significantly higher risk of sarcopenia (SPPB ≤ 9) compared to patients with a normal PS (0–1). However, HGS (<10th percentile), which is also a surrogate measurement for sarcopenia, was not significantly associated with a poor PS (2–3). Risk of frailty (MFST-HP ≥ 6) was not significantly associated with PS, although patients with a poor PS (2–3) had a significantly higher median score on MFST-HP than patients with PS (0–1) (Table 3). 

Advanced cancer stage grouping (3–4) was not significantly associated with risk of malnutrition (SNAQ > 2 or BMI < 20 kg/m^2^ if age < 70 y and <22 kg/m^2^ if age ≥ 70 y) nor with the measurement of frailty using the MFST-HP > 6. However, advanced cancer stage grouping (3–4) was significantly associated with a higher risk of OD (EAT-10 > 3) and high levels of distress (DT > 5) compared to early cancer stage grouping (0–2). Regarding the measurements of risk of sarcopenia, while median SPPB was significantly associated with advanced cancer stage grouping (3–4), an SPPB ≤ 9, median HGS, and HGS < 10th percentile were not (Table 3). 

The results of the multivariable logistic regression analysis are presented in Table 4. No significant effects were observed regarding the association between age (>70 years versus ≤70 years) and increased risk of the four screening phenomena after correction for advanced cancer stage grouping only and after additional correction for tobacco and alcohol consumption. 

Advanced cancer stage grouping (3–4) was significantly related to increased risk of OD (EAT-10 ≥ 3) and high levels of distress (DT ≥ 5) after correction for age. After additional correction for tobacco and alcohol consumption, respectively, both the risk of OD (EAT-10 ≥ 3) and high levels of distress (DT ≥ 5) remained significantly associated with advanced cancer stage grouping (3–4). 

The sensitivity analysis using linear regression analysis with correction for cancer stage grouping, tobacco, and alcohol consumption (Supplementary Table S1) showed that age > 70 years remained significantly associated with sarcopenia (SPPB). After correction for age and additional correction for tobacco and alcohol consumption, the sensitivity analysis also showed a significant association between advanced cancer stage grouping (3–4) versus OD (EAT-10), malnutrition (SNAQ), and levels of distress (DT). Neither evidence of multicollinearity nor of influential outliners was observed.

## 4. Discussion

The present study identified the prevalence of the risk of OD, malnutrition, sarcopenia, and frailty and investigated the relationship between these four phenomena versus age, PS, and cancer stage grouping in newly diagnosed HNC patients using a standardized multi-domain screening. 

In an ideal world, every newly diagnosed HNC patient should receive a CGA to identify a patient’s risk profile prior to cancer treatment. A meta-analysis by Cleere et al. concluded that 5.4 to 52.3% of the HNC patients were at risk of frailty [7]. A CGA is considered the gold standard for identifying frail patients with HNC and focuses on multiple dimensions of frailty, including medical, functional, psychological, and nutritional limitations [26,27]. While several CGA tools, such as the modified Frailty Index (mFI), the Groningen Frailty Indicator (GFI), and the G8 questionnaire [64,65,66], were designed for the older cancer population, they are not sufficiently specific for the limitations of the HNC population. For example, assessment of swallowing impairment is lacking in these CGA tools, which is a critical domain for the HNC population [26]. Furthermore, a systematic assessment of every newly diagnosed HNC patient by a team of health professionals requires at least 1 h consultations, making it infeasible to refer every newly diagnosed HNC patient for a CGA [30]. Given the lifestyle problems and the known risk of comorbid conditions in this population, biological and calendar age will often be discordant, which means that all newly diagnosed HNC patients are eligible for a CGA. Therefore, in close collaboration with the interdisciplinary HNC team of the MUMC+, we developed a multi-domain screening for everyday practice that can be performed by trained oncology nurses. This multi-domain screening provides quick identification of a patient’s risk profile with clinically relevant domains for newly diagnosed HNC patients. Patients with positively screened domains can subsequently be referred to designated experts for in-depth diagnostic assessment and treatment of a particular condition. The multi-domain screening in the current study took, on average, 15 min per patient, including an explanation of the results and referral to specific allied health and/or medical disciplines.

Approximately a quarter of the patients were at risk of OD (EAT-10 ≥ 3) prior to cancer treatment. A recent study on the association between EAT-10 scores pre- and post-cancer treatment reported that 58% of the newly diagnosed HNC patients scored EAT-10 ≥ 3 [34]. This high percentage emphasizes the importance of screening for OD as part of a frailty assessment or risk profiling. Compared to the present study, this single-domain study on the risk of OD included only patients undergoing chemoradiation who had an advanced tumor classification (T2–4). Interestingly, a pre-cancer treatment EAT-10 score ≥ 3 was associated with bolus aspiration and tube feeding dependency after cancer treatment, supporting our idea that screening for risk of OD in newly diagnosed HNC patients is important for early initiation of swallowing (pre)habilitation and for subsequent screening of malnutrition [34]. 

Moreover, in the present study, one-third of the newly diagnosed HNC patients were at risk of malnutrition. Several validated screening tools were used in previous studies to determine the risk of malnutrition in HNC patients, and these studies reported that 28.1% to 39% of newly diagnosed HNC patients had a higher risk of malnutrition [35,36,37]. The authors concluded that malnutrition risk and frailty are coexisting but distinct conditions for which screening is indicated in HNC patients [35]. Screening for risk of OD and malnutrition in all newly diagnosed HNC patients is inevitable, as OD and malnutrition can also have clinically significant consequences such as aspiration pneumonia, reduced tolerance to cancer treatment, reduced survival, and poor health-related quality of life [67,68]. 

Next, three quarters of the patients were at risk of sarcopenia in the present study. A recent meta-analysis reported on the assessment of sarcopenia in newly diagnosed HNC patients using the golden standard methods based on magnetic resonance imaging (MRI) or computed tomography (CT) scans. This meta-analysis reported that 6.6 to 64.6% of the newly diagnosed HNC patients had a condition of sarcopenia [16]. MRI- or CT-derived measurements of body composition and sarcopenia are still labor-intensive and therefore expensive [15]. Furthermore, in the present sample of HNC patients, abdominal CT scans, including the level of the third lumbar vertebrae being the most accurate reference site to assess whole-body muscle mass, were not carried out as this examination is not part of standard care for HNC patients in the Netherlands. Instead of these CT-derived diagnostic sarcopenia measurements, the risk of sarcopenia in the present study population was screened using the BMI, the SPPB, and HGS according to the recommendations of the EWGSOP2 [15]. A high risk of sarcopenia based on these surrogate markers is an indication of sarcopenia treatment [15]. According to our knowledge, the study of Willemsen et al. is the first to examine the relationship between cancer-related loss of skeletal muscle- and adipose tissue versus patient-reported OD in HNC patients prior to cancer treatment [69]. Both patient-reported OD using the EAT-10 and cancer-related loss of skeletal muscle mass and muscle function using, among others, the BMI, SPPB, and HGS were co-occurring conditions in HNC patients prior to cancer treatment. Nearly forty percent of the population in the study by Willemsen et al. reported clinically relevant symptoms of OD (EAT-10 ≥ 3), and twenty-six percent of the total population was considered cachectic. In contrast to our study, this study only looked at HNC patients with advanced cancer stage grouping who had to undergo chemoradiation or bioradiation, so a lower prevalence of OD and sarcopenia was expected in the present study. 

The prevalence of positively screened phenomena is high in the current heterogeneous study population consisting of newly diagnosed HNC patients. The second research question was therefore whether screening can be performed even more efficiently if it appears that there are associations between positively screened phenomena versus age, PS, and/or cancer group staging. In other words, the screening could be more focused on subgroups of newly diagnosed HNC patients, such as those patients with higher age, lower PS, or higher cancer group staging. As stated before, age, PS, and cancer stage grouping are major criteria used in cancer treatment decision-making as they are important predictors of tolerance to treatment and survival [38,39,40]. Using logistic regression analysis, age > 70 years was not identified as an indicator for positive risk screening in any domain. This is an important finding when we realize that CGA and assessment of sarcopenia prior to cancer treatment are usually performed in the older cancer population [26,70].

However, an association was found between poor PS and risk of sarcopenia, which seems logical from a clinical point of view since patients with low physical activity have less ability to activate muscle stem cells and therefore show a higher risk of sarcopenia [71]. Although no significant association was found between poor PS versus the risk of high levels of distress and frailty, an association was found between poor PS versus median DT and median MFST-HP scores. This finding is probably due to the low number of patients with poor PS (N = 14). Due to this low subgroup sample size, we were not able to determine the associations between poor PS and risk of the other screened phenomena after correction for age, tobacco, and alcohol consumption. 

Furthermore, advanced cancer stage grouping remained an indicator for the presence of risk of OD and high levels of distress in the multivariable regression analysis with correction for age, tobacco, and alcohol consumption. It was expected that advanced cancer stage grouping was an indicator of the presence of OD since the more advanced disease can cause upper digestive tract dysfunction by the primary tumor itself and/or extensive neck node metastases, invading important swallowing structures such as muscles and cranial nerves [10]. Despite the absence of an association between advanced cancer stage grouping and sarcopenia in the present study, it has been described that cancer metabolism-induced skeletal muscle wasting due to high catabolic activity is more pronounced in advanced disease stages [15]. These catabolic processes promoting skeletal muscle wasting will more than likely affect the muscles involved in swallowing, leading to OD. Currently, high-quality scientific evidence to confirm the more than likely causal relationship between OD and catabolic muscle wasting is lacking. However, the presence of OD in newly diagnosed patients with other non-HNC tumor sites, such as lung and gastro-intestinal cancers, makes this assumption more plausible [67,72]. 

In summary, we can conclude that the risk of OD, malnutrition, sarcopenia, and frailty are probably partly overlapping phenomena, but they are by no means concordant or synonymous. SPPB provides information on physical disability and sarcopenia, with sarcopenia being a risk factor and physical disability being an outcome of frailty [73,74]. In newly diagnosed HNC patients, concurrent screening for OD, malnutrition, sarcopenia, and frailty can provide important complementary information about the patient’s risk profile. In this way, we can, to a certain extent, include a patient’s baseline risk profile in shared decision making, prehabilitation, and a patient-centered selection of cancer treatment modalities. Finally, the prevalence of being at risk for other phenomena besides frailty was high in the current study, warranting multi-domain screening in all HNC patients prior to cancer treatment. Future studies are needed to determine the effects of multi-domain screening on long-term cancer outcomes, such as health-related quality of life and survival. 

### Limitations

The present prospective study has methodological limitations. The study revealed some interesting statistically significant preliminary data. However, the sample size may be too small to reveal all significant associations or group differences. The heterogeneous etiology of cancer anatomical sites and histopathology and the small number of patients per group made it impossible to compare all subgroups. Because of the low subgroup size of the dichotomized PS, HGS, and MFST-HP, these screening variables could not be included in the multivariable logistic regression analysis.

Furthermore, there are other screening tools for our multi-domain phenomena available [35,36,37,64,65,66]. Another tool may have produced different results in the present study. However, the process leading to the selection and implementation of the screening tools in everyday practice is described in the Supplementary Table S2, and there is evidence in the literature, as described in the paragraph discussion, to support the selection of the screening tools in the current study. 

## 5. Conclusions

This study identified the risk profile of newly diagnosed HNC patients using a standardized ‘quick and easy’ multi-domain screening prior to cancer treatment. More than three quarters of the 128 newly diagnosed HNC patients were at risk of OD, malnutrition, sarcopenia, and/or frailty. Patients with an advanced cancer stage had a significantly higher risk of OD and high levels of distress prior to cancer treatment. If only screened for frailty, the number of patients at risk is approximately one out of ten. The prevalence of being at risk for other phenomena besides frailty was much higher than one in ten, warranting multi-domain screening in all HNC patients prior to cancer treatment. 

## Figures and Tables

**Table 1:** Patient demographic data, tumor characteristics, CCI grades, and PS of patients who underwent multi-domain screening.

Variable	Patients (N = 128)
Age, mean (SD) (N = 128)	67 (9.8)
Age ≤ 70 years, N (%)	81 (63.3)
Age > 70 years, N (%)	47 (36.7)
Sex, N (%)	128 (100.0)
Male	84 (65.6)
Female	44 (34.4)
Tobacco consumption, N (%)	128 (100.0)
Never	20 (15.6)
Former	57 (44.5)
Current	51 (39.8)
Number of pack years of smoking, median (IQR) (N = 128)	28 (5–45)
Alcohol consumption, N (%)	127 (100.0)
Never or irregular drinker (<one alcoholic drink per day)	69 (54.3)
Former drinker (at least one alcoholic drink per day in the past)	10 (7.9)
Current regular drinker (at least one alcoholic drink per day)	48 (37.8)
Number of alcoholic drinks per day, median (IQR) (N = 127)	1 (0–3)
Marital status, N (%)	126 (100.0)
Single	23 (18.3)
Married	81 (64.3)
Widower	14 (11.1)
Having a partner without being married	8 (6.3)
Occupation, N (%)	91 (100.0)
Employed	35 (38.5)
Unemployed	15 (16.5)
Retired	38 (41.8)
Voluntary work	3 (3.3)
Tumor site, N (%)	128 (100.0)
Oral cavity	46 (35.9)
(Para)nasal cavity	8 (6.3)
Pharynx	39 (30.5)
Larynx	28 (21.9)
Salivary gland	4 (3.1)
Lymph node metastasis of unknown origin	3 (2.3)
T classification, N (%)	128 (100.0)
T0–1	56 (43.8)
T2	27 (21.1)
T3	22 (17.2)
T4	23 (18.0)
N classification, N (%)	128 (100.0)
N0	82 (64.1)
N1	19 (14.8)
N2	21 (16.4)
N3	6 (4.7)
M classification, N (%)	128 (100.0)
M0	127 (99.2)
M1 ^a^	1 (0.8)
Cancer stage grouping, N (%)	128 (100.0)
0–1	55 (43.0)
2	19 (14.8)
3	19 (14.8)
4	35 (27.3)
Tumor histopathology, N (%)	128 (100.0)
Squamous cell carcinoma	115 (89.8)
Verrucous carcinoma	4 (3.1)
Other histopathology	9 (7.0)
HPV/p16 positive, N (%) (N = 128)	16 (12.5)
EBV positive, N (%) (N = 128)	2 (1.6)
CCI grade, N (%)	128 (100.0)
0 No comorbidity	52 (40.6)
1 Low-level comorbidity	51 (39.8)
2 Moderate-level comorbidity	16 (12.5)
3 Advanced-level comorbidity	9 (7.0)
PS, N (%)	128 (100.0)
0 Asymptomatic	90 (70.3)
1 Symptomatic, fully ambulatory	24 (18.8)
2 Symptomatic, in bed <50% of the day	12 (9.4)
3 Symptomatic, in bed >50% of the day but not bedridden	2 (1.6)
4 Completely disabled, bedridden	0 (0.0)

Abbreviations: HNC—Head and Neck Cancer; N—Number of patients; SD—Standard Deviation; IQR—Interquartile Range; HPV—Human papillomavirus; EBV—Epstein–Barr virus; CCI—Charlson Comorbidity Index; PS—WHO performance status. ^a^ Patient underwent curative cancer treatment with an oligometastatic protocol.

**Table 2:** Frequency distribution, measures of central tendency, and dispersion of the multi-domain screening.

Multi-Domain Screening	Patients (N = 128)
**Domain OD**	
EAT-10, N (%)	126 (100.0)
EAT-10 median (IQR)	0 (0–3)
<3, N (%)	93 (73.8)
≥3, N (%)	33 (26.2)
**Domain malnutrition**	
SNAQ, N (%)	124 (100.0)
SNAQ median (IQR)	0 (0–1)
<2, N (%)	99 (79.8)
≥2, N (%)	25 (20.2)
BMI, N (%)	128 (100.0)
BMI mean (SD)	25.8 (5.0)
≥20 kg/m^2^ if age < 70 y and ≥22 kg/m^2^ if age ≥ 70 y, N (%)	108 (84.4)
<20 kg/m^2^ if age < 70 y and <22 kg/m^2^ if age ≥ 70 y, N (%)	20 (15.6)
**Domain sarcopenia**	
SPPB, N (%)	73 (100.0)
SPPB median (IQR)	9 (8–10)
>9, N (%)	22 (30.1)
4–9, N (%)	48 (65.8)
<4, N (%)	3 (4.1)
HGS ^a^, N (%)	71 (100.0)
≥10th percentile, N (%)	62 (87.3)
<10th percentile, N (%)	9 (12.7)
**Domain frailty**	
DT, N (%)	125 (100.0)
DT median (IQR)	4 (1–7)
<5, N (%)	67 (53.6)
≥5, N (%)	58 (46.4)
MFST-HP ^b^, N (%)	128 (100.0)
MFST-HP median (IQR)	2 (1–3)
<6, N (%)	35 (89.7)
≥6, N (%)	4 (10.3)

Abbreviations: HNC—Head and Neck Cancer; N—Number of patients; SD—Standard Deviation; IQR—Interquartile Range; OD—Oropharyngeal dysphagia; EAT-10—Eating Assessment Tool; SNAQ—Short Nutritional Assessment Questionnaire; BMI—Body Mass Index; SPPB—Short Physical Performance Battery; HGS—Hand Grip Strength; DT—Distress thermometer; MFST-HP—Maastricht Frailty Screening Tool. ^a^ HGS could not be presented in a numerical way (median, IQR) because HGS percentiles were based on sex-, age-, and height-appropriate references which are reported in a categorical manner rather than continuously [58]. ^b^ Only patients aged above 70 years and patients younger than 70 with a severe-comorbid condition.

**Table 3:** Group comparisons of multi-domain screening results of newly diagnosed HNC patients with age < 70 versus age > 70, PS 0–1 versus PS 2–3, and patients with CSG 0–1 versus CSG 3–4.

Multi-Domain Screening	Age ≤ 70 Years (N = 81)	Age > 70 Years (N = 47)	*p*-Value	PS 0–1 (N = 114)	PS 2–3 (N = 14)	*p*-Value	CSG 0–2 (N = 74)	CSG 3–4 (N = 54)	*p*-Value
**Domain OD**									
EAT-10 (N = 126)									
EAT-10 median (IQR)	0 (0–2)	0 (0–5)	0.400 ^a^	0 (0–2)	0 (0–8)	0.664 ^a^	0 (0–1)	1 (0–12)	**0.001** ^a^
≥3, N (%)	19 (23.8)	14 (30.4)	0.528 ^b^	28 (25.0)	5 (35.7)	0.519 ^c^	8 (11.1)	25 (46.3)	**<0.001** ^b^
**Domain malnutrition**									
SNAQ (N = 124)									
SNAQ median (IQR)	0 (0–0)	0 (0–2)	**0.032** ^a^	0 (0–1)	0 (0–1)	0.507 ^a^	0 (0–0)	0 (0–2)	**0.014** ^a^
≥2, N (%)	12 (15.4)	13 (28.3)	0.106 ^b^	22 (20.0)	3 (21.4)	1.000 ^c^	11 (15.1)	14 (27.5)	0.113 ^b^
BMI (N = 126)									
BMI mean (SD)	26.1 (5.3)	25.5 (4.5)	0.518 ^d^	25.7 (4.8)	26.9 (6.6)	0.427 ^d^	26.0 (5.3)	25.5 (4.6)	0.469 ^d^
<20 kg/m^2^ if age < 70 y and <22 kg/m^2^ if age ≥ 70 y, N (%)	10 (12.3)	10 (21.3)	0.211 ^b^	18 (15.8)	2 (14.3)	1.000 ^c^	10 (13.5)	10 (18.5)	0.677 ^c^
**Domain sarcopenia**									
SPPB (N = 73)									
SPPB median (IQR)	9 (9–10)	8 (5–9)	**0.001** ^a^	9 (8–10)	6 (5–8)	**<0.001** ^a^	9 (9–10)	9 (7–9)	**0.031** ^a^
≤9, N (%)	31 (62.0)	20 (87.0)	0.053 ^b^	41 (65.1)	10 (100.0)	**0.027** ^c^	25 (61.0)	26 (81.3)	0.075 ^b^
HGS (N = 71)									
<10th percentile, N (%) ^e^	5 (10.4)	4 (17.4)	0.458 ^c^	6 (9.8)	3 (30.0)	0.108 ^c^	5 (11.9)	4 (13.8)	1.000 ^c^
**Domain frailty**									
DT (N = 125)									
DT median (IQR)	4 (1–7)	3 (1–7)	0.751 ^a^	3 (1–7)	7 (2–8)	0.076 ^a^	2 (0–7)	6 (2–7)	0.003 ^a^
≥5, N (%)	38 (46.9)	20 (45.5)	1.000 ^b^	49 (44.1)	9 (64.3)	0.169 ^b^	27 (38.0)	31 (57.4)	**0.046** ^b^
MFST–HP (N = 39)									
MFST-HP median (IQR)	2 (2–2)	2 (1–3)	0.892 ^a^	2 (1–3)	4 (2–9)	**0.015** ^a^	2 (1–4)	2 (1–3)	0.728 ^a^
≥6, N (%)	0 (0.0)	4 (10.5)	1.000 ^c^	2 (6.1)	2 (33.3)	0.104 ^c^	3 (14.3)	1 (5.6)	0.609 ^c^

Abbreviations: HNC—Head and Neck Cancer; N—Number of patients; SD—Standard Deviation; IQR—Interquartile Range; PS—Performance status; CSG—Cancer stage grouping; OD—Oropharyngeal dysphagia; EAT-10—Eating Assessment Tool; SNAQ—Short Nutritional Assessment Questionnaire; BMI—Body Mass Index; SPPB—Short Physical Performance Battery; HGS—Hand Grip Strength; DT—Distress thermometer; MFST-HP—Maastricht Frailty Screening Tool. ^a^ Mann–Whitney U test. ^b^ Pearson’s chi-square test (χ^2^).^c^ Fisher’s exact test. ^d^ Independent samples *t* test. ^e^ HGS could not be presented in a numerical way (median, IQR) because HGS percentiles were based on sex-, age-, and height-appropriate references, which are reported in a categorical manner rather than continuously [58].

**Table 4:** Multivariable logistic regression analysis showing predictors of the risk of OD, malnutrition, sarcopenia, and frailty.

Variables	Age > 70 Years Versus Age ≤ 70 Years (after Correction for CSG)		Age > 70 Years Versus Age ≤ 70 Years (after Correction for CSG and Tobacco and Alcohol Consumption)		CSG 3–4 Versus CSG Grouping 1–2 (after Correction for Age)		CSG 3–4 Versus CSG 1–2 (after Correction for Age and Tobacco and Alcohol Consumption)	
	**OR (95% CI)**	***p*** **-Value**	**OR (95% CI)**	***p*** **-Value**	**OR (95% CI)**	***p*** **-Value**	**OR (95% CI)**	***p*** **-Value**
**Domain OD**								
EAT-10 (≥3) (N = 33)	1.3 (0.5–3.2)	0.531	1.3 (0.5–3.3)	0.546	6.5 (2.6–16.2)	**<0.001**	6.9 (2.7–17.4)	**<0.001**
**Domain malnutrition**								
SNAQ (≥2) (N = 25)	2.3 (0.9–5.7)	0.078	2.3 (0.9–5.8)	0.075	1.9 (0.8–4.7)	0.173	1.9 (0.8–4.7)	0.179
BMI (<20 kg/m^2^ if age < 70 y and <22 kg/m^2^ if age ≥ 70 y) (N = 20)	2.1 (0.8–5.6)	0.141	2.1 (0.8–5.8)	0.142	1.2 (0.5–3.3)	0.672	1.3 (0.5–3.5)	0.651
**Domain sarcopenia**								
SPPB (≤9) (N = 51)	3.5 (0.9–13.6)	0.075	3.5 (0.9–14.1)	0.075	2.3 (0.7–7.0)	0.149	2.2 (0.7–6.8)	0.180
**Domain frailty**								
DT (≥5) (N = 58)	0.9 (0.4–1.9)	0.752	0.9 (0.4–1.9)	0.718	2.2 (1.04–4.5)	**0.039**	2.2 (1.02–4.6)	**0.044**

Abbreviations: OD—Oropharyngeal dysphagia; CSG—cancer stage grouping; N—Number of patients; OR—Odds Ratio; CI—Confidence Interval; EAT-10—Eating Assessment Tool; SNAQ—Short Nutritional Assessment Questionnaire; BMI—Body Mass Index; SPPB—Short Physical Performance Battery; DT—Distress thermometer.

## Data Availability

The data that support the findings of this study are available upon request from the corresponding author. The data are not publicly available due to privacy restrictions.

## Supplementary Materials

The following supporting information can be downloaded at https://www.mdpi.com/article/10.3390/cancers15215254/s1. Table S1: Sensitivity analysis using multivariable linear regression analysis showing predictors of the risk of OD, malnutrition, sarcopenia, and frailty; Table S2: Justification for the selection of the screening tools used in the current study.
