# Supplementary material for: Multi-Domain Screening: Identification of Patient’s Risk Profile Prior to Head-and-Neck Cancer Treatment
Source: Cancers (Basel). 2023 Nov 1;15(21):5254. doi: 10.3390/cancers15215254 (PMC10648822; doi:10.3390/cancers15215254)
Supplement: Supplementary file 1 [file cancers-15-05254-s001.zip › cancers-2671941-supplementary.pdf]

cancers MDPI

Supplementary material

Table S1. Sensitivity analysis using multivariable linear regression analysis showing predictors of the risk of OD, malnutrition, sarcopenia, and frailty.

Variables	Age > 70 years versus age ≤ 70 years (after correction for CSG)		Age > 70 years versus age ≤ 70 years (after correction for CSG, and tobacco and alcohol consumption)		CSG 3-4 versus CSG grouping 1-2 (after correction for age)		CSG 3-4 versus CSG 1-2 (after correction for age, and tobacco and alcohol consumption)	
	**B (SE)**	**p-value**	**B (SE)**	**p-value**	**B (SE)**	**p-value**	**B (SE)**	**p-value**
**Domain OD**								
EAT-10	1.5 (1.1)	0.375	1.1 (2.0)	0.592	4.7 (1.4)	**0.001**	4.5 (1.5)	**0.003**
**Domain malnutrition**								
SNAQ	0.4 (0.3)	0.128	0.5 (0.3)	0.100	0.6 (0.3)	**0.030**	0.5 (0.3)	**0.047**
BMI	-0.6 (0.9)	0.544	-0.6 (0.9)	0.491	-0.5 (0.9)	0.608	-0.3 (0.9)	0.733
**Domain sarcopenia**								
SPPB	-1.9 (0.6)	**0.001**	-2.0 (0.6)	**0.001**	-0.7 (0.5)	0.208	-0.7 (0.6)	0.226
**Domain frailty**								
DT	-0.4 (0.6)	0.519	-0.4 (0.6)	0.536	1.7 (0.6)	**0.003**	1.6 (0.6)	**0.005**

Abbreviations: OD - Oropharyngeal dysphagia; CSG – cancer stage grouping; B – Regression coefficient; SE – Standard Error; EAT-10 - Eating Assessment Tool; SNAQ - Short Nutritional Assessment Questionnaire; BMI - Body Mass Index; SPPB - Short Physical Performance Battery; DT - Distress thermometer.

Table S2. Justification for the selection of the screening tools used in the current study.

**Justification for the selection of the screening tools used in the current study.**
The process leading to the selection of the screening tools for the multi-domain screening was as follows. In 2019, the head and neck oncology care line at the Comprehensive Cancer Center MUMC+ started monthly meetings with HNC-dedicated health professionals delegated by each allied health and/or medical discipline and with HNC-dedicated researchers. The purpose of these meetings was to identify how HNC patients were screened for multiple risk domains from arrival – prior to cancer treatment – and following cancer treatment until the end of the oncological follow-up trajectory. What was the indication for referral to a specific allied health and/or medical discipline, who determined this referral indication, what was screened prior to referral, who screened and how often, what was the next step in terms of in-dept diagnostics if the screening was abnormal, etc. After an inventory of these 'habits' or standards, it was examined per discipline which screening instruments were used and whether these were evidence-based or whether they should be replaced by an instrument with better psychometric properties for this specific patient population. This process took about one year and based on available scientific evidence and consensus discussion by the HNC-dedicated delegates, the multi-domain screening was established and implemented in everyday practice. Three oncology nurses were trained and supervised by two HNC-dedicated researchers and a head-and-neck surgeon to carry out the multi-domain screening. This study was also developed in collaboration with two patients from a dedicated specialized patient involvement group (Patiëntenvereniging Hoofd-Hals/PVHH). Both patients are HNC survivors and the PVHH endorsed the current study project.
